# Mercury, Blue Pill, and Calomel, Their Use and Abuse

**Published:** 1840-07

**Authors:** 


Art. IV. *Mercury, Blue Pill, and Calomel, their use and abuse.* By George G. Sigmond, m.d., f.s.a., f.l.s., &c.—*London,* 1840. 12mo, pp. 229.

This little work, we are told in the preface, is a reprint of some lectures that originally appeared in one of our medical Journals. We hardly think these need have been disturbed in their repose; yet they are not without some value. The first 26 pages of the volume are occupied with a general history of the metal and its applications, written in a very lively and amusing though not always correct style. The author then describes the various preparations of mercury in the pharmacopœia, and points out the diseases to which they are severally most applicable: he also comments on the use of other remedies that are usually combined with mercury in the treatment of the diseases noticed, or that have obtained celebrity in such cases upon their own merits. From among some good practical hints scattered up and down this part of the work, we shall select a few observations upon the abuse of purgatives, by way of an appendix to our article on Dr. Burne's book in another part of the present Number: they unfold "an ower true tale," not a little discreditable to British therapeutics.

"When a medical man is called in, who is an advocate for the purging system, patients generally soon fall into his way of thinking; they have a sort of gratification in discharging a large quantity of nasty-looking greenish or black stuff. They fancy they have discharged a mass of corruption; and they are quite ready for another dose of blue pill and a draught.... They are very much pleased with what has been done for them; and, indeed, were they to rest contented all would be well; but they fancy they have discovered the means of attaining lasting health, they therefore give way to the pleasures of the table, quite satisfied that they know how to get rid of any accumulation that may occur.... Sometimes, after dinner, a kind friend recommends his own pill from a prescription he had from Abernethy or Baillie, or the fashionable dyspeptic doctor of the day, which he declares he has used twice a week for the last five years, and never known an ache or pain since; the greedy auditor swallows the tale, and afterwards the physic, whose composition may be completely opposed to his state: he goes on with it, week after week, disordering his bowels, expelling the natural mucus, urging on the canal to increased action, till at last his stomach and the whole digestive apparatus become really impaired. He then goes from physician to physician all over London; each gives him a prescription for a tonic, a stimulant, or a bitter; at length a permanent irritation is kept up, the whole system sympathises with the alimentary canal, the mind is feverish and irritable, the viscera become deranged, and at last the poor sufferer falls a victim to the early benefit which he had received from the proper administration of medicine, and then from its abuse." (p. 67.)

"The unnatural colour and the fœtid odour of the stools are quite as often produced by the medicine that has been exhibited as by any other cause, for often does calomel give rise to the very odour which some persons think is a proof that medicine is to be continued; the slimy stools of children, which are often the cause of purging being carried on to a very great extent are, in many instances, the consequence of what has been given. As long as the remedial agent is taken the fæcal residue does not acquire its wonted solid consistence, nor give forth the usual smell, which is acquired from its remaining in a certain portion of the large intestines......The offensiveness of the fæcal evacuation, though it may guide the practitioner as to the particular viscus that is disordered, cannot serve as any assistant by which he may ascertain whether more action is required...... Although in many diseases of infancy and of childhood aperient medicine is certainly useful, yet it should ever be borne in mind, that with excrementitious matter, there may be very often carried away that which nature has with some difficulty elaborated for nutrition; and in weak sickly children who have a tendency to scrofula, it not unfrequently happens that the food which was intended to be imbibed is just carried away at the moment when the glands most require something to store up for future nourishment." (p. 69.)

Dr. S. is opposed to minute doses of mercury, because they produce the same irritation as large ones, and are not carried off by the bowels. But he is loud in his praises of sarsaparilla. He gives a theory of the mode of action of this remedy which may be true, though we think not, and which we shall not therefore transcribe. We however concur with him in his high estimation of sarsaparilla after a mercurial course and indeed in conjunction with it.

